# Local infiltration with cocktail analgesics during 2 level lumbar spinal fusion surgery: Study protocol of a randomized controlled trial

**DOI:** 10.1097/MD.0000000000015526

**Published:** 2019-05-13

**Authors:** Zhinan Ren, Zheng Li, Shugang Li, Lin Sheng, Derong Xu, Xin Chen, William Ka Kei Wu, Matthew T.V. Chan, Jeffery Ho

**Affiliations:** a Department of Orthopaedics, Peking Union Medical College Hospital, Chinese Academy of Medical Sciences and Peking Union Medical College, Beijing; b Department of Orthopaedics, The First Affiliated Hospital Of Zhengzhou University, Zhengzhou; c Department of Anaesthesia and Intensive Care; d State Key Laboratory of Digestive Disease, LKS Institute of Health Sciences, The Chinese University of Hong Kong, Hong Kong, China.

**Keywords:** cocktail analgesia, lumbar spinal fusion, multimodal pain control, postoperative pain management

## Abstract

**Background:**

Despite introducing novel analgesics, pain management for spine surgery remains a challenge. Multimodal pain control has recently gained popularity in surgical spine care. We proposed a novel management approach using multimodal cocktail analgesics. Injection to skin surrounding surgical incision site will be given perioperatively. This study evaluates the safety and efficacy of cocktail analgesic injection on pain management following lumbar spinal fusion surgery.

**Methods:**

Thirty-six patients with degenerative lumbar spinal diseases on the waiting list for lumbar spinal fusion surgery will be recruited. Patients will be randomly assigned to receive either cocktail analgesic injection or sterile saline before surgical wound closure. All patients will routinely receive postoperative intravenous patient-controlled analgesia (IV-PCA) with sufentanil on an as-needed basis without a basal dose. The primary outcome is perceived pain intensity as measured by visual analog pain score. Secondary outcomes include sufentanil consumption, time to first use of IV-PCA, rescue analgesics consumption, and the presence of adverse effects. Findings of this interventional trial will provide novel evidence supporting the superior effect of cocktail analgesic injection during surgery.

**Trial registration number:**

ChiCTR-IPR-17013094.

## 1 Introduction

Patients undergone major spine surgery frequently experience severe pain after operation, lasting for more than 3 days.^[1]^ The underlying causes include direct surgical trauma^[2]^ and activation of multiple cellular targets pertinent to nociceptive, and inflammatory pathways.^[3]^ Peripheral and central sensitization further aggravates postoperative pain. Postoperative pain reduces patients’ satisfaction and substantially affects recovery, increases postoperative morbidities, and prolongs the length of hospital stay.^[4]^

Adequate postoperative pain management is essential to improve functional outcome, accelerate early ambulation, shorten the duration of hospital stay, and prevent subsequent development of chronic postsurgical pain. Different postoperative analgesics are available in various forms of administrative routes. Mechanisms contributing to postoperative pain in spine surgery are multifactorial. In this connection, single analgesic may be inadequate to achieve desirable outcomes. Compared with conventional analgesia, multimodal analgesia has recently gained popularity. This therapeutic approach combines different analgesics with multiple routes of administration to better optimize pain relief and minimize undesirable side effects and complications.^[5]^ While epidural and intravenous routes of drug administration may achieve analgesia,^[6–8]^ these costly procedures demand specialized skills and may confer nausea, vomiting, pruritus and urinary retention, respiratory depression, and neuraxial hematoma.^[9–11]^ In contrary, administration of local wound injection surrounding incision does not require highly trained personnel. This localized injection also facilitates drug delivery to the area of surgical wound and minimize systemic exposure.^[12–14]^ We propose a novel multimodal analgesic regimen containing several medications, designated cocktail analgesics, which are injected surrounding the surgical incision perioperatively. This study aims to evaluate the efficacy of cocktail analgesics for postoperative pain management amongst patients having 2-levels underwent lumbar spinal fusion surgery. We will determine if cocktail analgesic injection would provide satisfactory postoperative analgesia.

## 2 Methods

### 2.1 Study design

**Figure 1:**
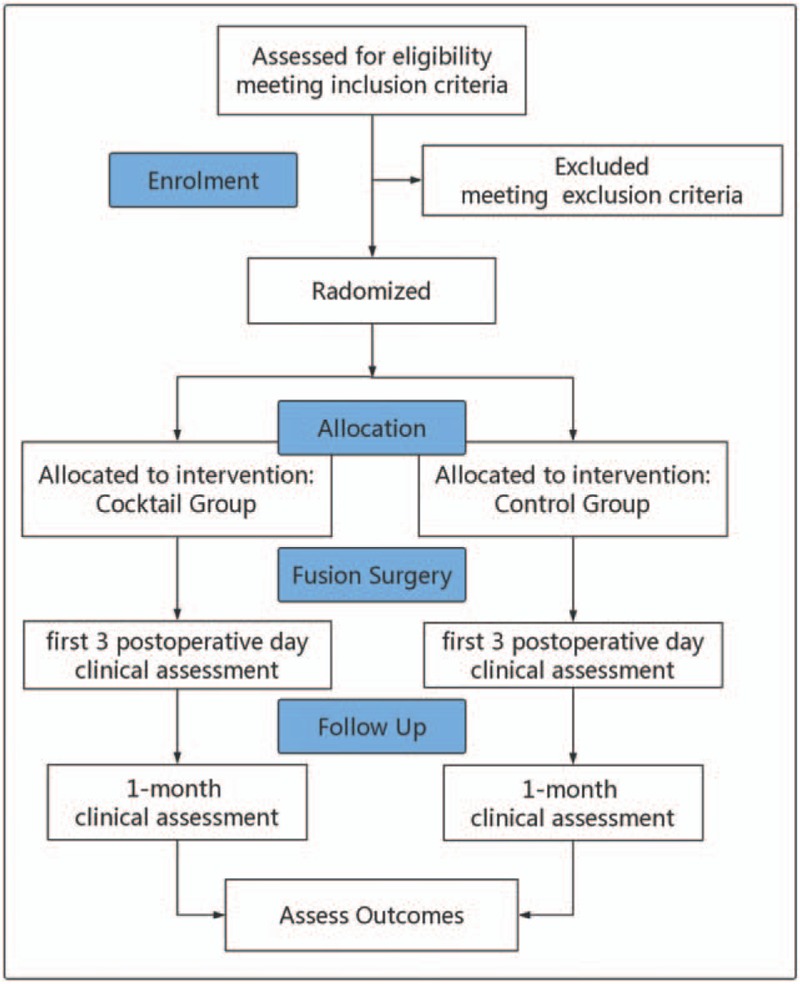
Trial flow diagram of the study.

This is a single-center, prospective, double-blinded, randomized controlled trial. The protocol will be performed in accordance with the Declaration of Helsinki and was approved by the Ethics Committee of Peking Union Medical College Hospital. Written informed consent will be obtained from each patient before enrollment. This trial is registered with the Chinese Clinical Trial Registry (ChiCTR-IPR-17013094). An overview of the trial design is shown in Figure 1.

### 2.2 Study center

Our center is a tertiary referral hospital for spine surgery with the necessary types and volume of clinical cases required for this study. The research team comprises a chief spine surgeon and a team of experienced surgeons, nurses and allied health staff.

### 2.3 Participants

Patients on the lumbar spinal fusions waiting list at Peking Union Medical College Hospital (PUMCH) will be invited to participate in this study. After initial screening, potentially eligible patients will be given a plain Chinese statement detailing the nature of the study and the commitment required. A trained researcher will obtain informed consent from eligible candidates who are willing to participate. Identified participants will be approached for inclusion in the study according to predefined inclusion and exclusion criteria.

### 2.4 Inclusion criteria

(1) Age between 18 and 70 years.
(2) On the surgical waiting list for primary 2-level lumbar spinal fusion surgery diagnosed with lumbar spinal stenosis or lumbar disc herniation.
(3) American Society of Anesthesiologists physical status I∼II.
(4) Understand and comply with the study protocol.

### 2.5 Exclusion criteria

(1) History of hypersensitivity to parecoxib, ropivacaine, diprospan, fentanyl, or sulfonamide.
(2) Perioperative period of coronary artery bypass surgery.
(3) Pregnant, lactating, or probably pregnant.
(4) Revision surgery or surgery for neoplastic disease.
(5) Inability to provide informed consent due to mental incompetence

### 2.6 Randomization

Random assignment will be performed before surgery using a computer-generated, block random-allocation sequence with a 1:1 ratio. Allocation concealment will be achieved using opaque sequentially numbered sealed envelopes. The research staff will record the patients’ details.

### 2.7 Blinding

The cocktail analgesic or equivalent volume of saline is prepared by pharmacy independent to the trial in an aseptic fashion. Both the attending surgeon and the patients will be blinded to the group allocation. Data will be collected by research staff blinded to group allocation. Upon completion of the study, a biostatistician blinded to group allocation will analyze outcome data.

### 2.8 Interventions

All patients will be randomized using a computer-generated list to 1 of 2 groups, 1 of which will receive subcutaneous injection of cocktail analgesic at final wound closure during surgery (cocktail group). Control group patients will receive equivalent volume of saline. The same surgeon will perform all operations. All patients will undergo 2-level posterior lumbar spinal fusions with pedicle screws and rod fixation.

### 2.9 Cocktail anesthetic preparation

Cocktail solution will contain ropivacaine 1%, 200 mg (20 ml), parecoxib 40 mg, betamethasone 7 mg (1 ml), mixed with normal saline into a total volume of 50 ml.

### 2.10 Cocktail analgesic injection group (cocktail group)

In the cocktail group, cocktail analgesics will be injected surrounding the deep fascia and the muscular layer in the surgical wound before final wound closure. A total of 35 ml trial solution will be used. Postoperative analgesia will be provided by intravenous patient-controlled analgesia (IV-PCA) delivering sufentanil 2 μg in each bolus, with a 15-minute lockout interval. No basal infusion will be used. PCA pump will be removed in 48 hours after surgery.

### 2.11 Sterile saline injection group (control group)

In the control group, an equal volume of saline (35 ml) will be injected to the deep fascia and the muscular layer in the surgical wound in the same fashion as the cocktail group. IV-PCA will be provided in the same mode.

### 2.12 Perioperative management

Surgery will be performed under general anesthesia with standardized fashion. A standardized intraoperative strategy for fluid management will be applied which consists of 0.9% saline at 5 ml/kg/h and hydroxyethyl starch colloid at 7.5 ml/kg/h. At the end of the surgery, the PCA pump will be started on without a basal dosage, which will be removed 48 hours later. After surgery, patients will be taken to the recovery room and monitored according to standard hospital policy. Patients will be educated with the use of the PCA device and discharged to the ward, once recovery room discharge criteria have been met. Patients will receive supplemental oxygen via nasal cannula at 2 l/min for 24 hours. The time to first mobilization will be recorded and the patient will continue to be monitored until discharge. Any serious adverse events (SAEs) will prompt follow up. Patients will be followed up routinely at 4 weeks after discharge by the spine specialist. Symptoms of nerve damage will be actively sought at this consultation.

### 2.13 Rescue analgesia

Patients who require an additional analgesic after PCA removal will be provided with tramadol 50 mg intramuscularly route each time. Usually, the need for supplementary treatment with systemic opioids is a reliable indicator when concerned about postoperative pain control. Having considered that the use of strong or long-term rescue analgesia in both groups may reduce pain too much and “washed out” any differences between the 2 groups. In this study, we use tramadol hydrochloride injection for rescue analgesia, which is a mid-intensity opioid analgesic that will facilitate observing the differences in pain control between groups. The frequency and total dosage during the first 3 postoperative days will be recorded.

### 2.14 Outcome assessments

#### 2.14.1 Data capture

Schedule for data collection is shown in Table 1. Specifically, the following data will be collected – demographic information, daily visual analog scale (VAS) pain score, sufentanil consumption, time to first use of IV-PCA, postoperative nausea and vomiting, rescue analgesia consumption, all other side effects and complications.

**Table 1:**
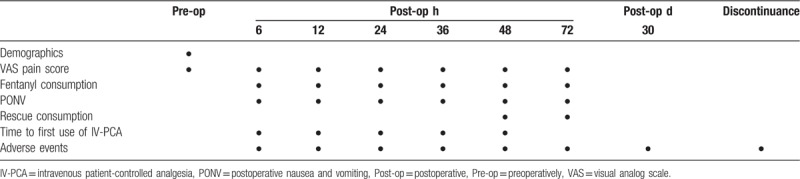
Data collection schedule.

The VAS has been used extensively for rating pain intensity in previous studies.^[15–17]^ Postoperative pain at 6, 12, 24, 36, 48, and 72 hours after surgery will be recorded using the VAS pain scores. The VAS consists of a 10-cm horizontal line with 1-cm vertical lines at each end on which a score of 0 indicates no pain and a score of 10 indicates the worst conceivable pain. All data are verified against original documents by individuals who are independent of the trial.

### 2.15 Primary outcome measures

The primary outcome was the average pain score over the incision site from the time of surgery up to 3 postoperative days after surgery

### 2.16 Secondary outcome measures

#### 2.16.1 Sufentanil consumption

Sufentanil consumption of IV-PCA during the first 48 hours postoperatively will be recorded.

#### 2.16.2 Time to first use of IV-PCA

The time to first use of IV-PCA and proportion of the patients during the first 48 hours postoperatively will be recorded.

#### 2.16.3 Rescue analgesia consumption

The frequency and total dosage of the rescue analgesics (tramadol hydrochloride injection) during the first 72 hours postoperatively will be recorded.

#### 2.16.4 Safety

The presence of side effects during the entire postoperative period will be recorded, including nausea, vomiting, and other adverse events and complications.

### 2.17 Sample size

The sample size calculation was based on the following parameters:

(1) alpha value = 0.05, 2-sided;
(2) power = 80%.

The minimal clinical significance in VAS pain score was defined as the mean difference between current and preceding scores when the subject reported “a little worse” or “a little better” pain. Our pilot study shows that VAS pain scores 72 hours after lumbar spinal fusion surgery were reported to be 2.1 ± 1.6 in patients receiving cocktail analgesic injection and 5.6 ± 1.7 in patients without. Based on these findings, we hypothesized that VAS pain score 72 hours after surgery will be 2.5 for the cocktail group and 5.0 for the control group, with a standard deviation set at 2.5 and a ratio of patients in the cocktail to the control group of 1:1. Calculations showed that a sample size of 14 patients per group to detect a difference in VAS pain score between the 2 groups after treatment. Assuming a 20% dropout rate, 18 patients per group, or a total of 36 patients, are required.

It is anticipated that recruitment for this study will take 6 months to complete, using 1 surgeon to reduce surgical variability. Data collection for each patient will occur during the first 72 hours postoperatively and at a routine 4-week follow-up appointment. No further follow up will be routinely arranged. Any patients requiring specific follow up will have this arranged on an individual basis.

### 2.18 Timeline

This is a 6-month study commencing January 2018 and ending June 2019. See Table 1 and Figure 1 for time points and recruitment progress.

### 2.19 Criteria for discontinuation

Every effort will be made to retain patients in the trial and to minimize withdrawals. However, the trial will be discontinued on condition that any SAEs happen. Additionally, patients may request to be withdrawn from this study at any time without any reason. Intention to treat and “as treated” analyses will be performed.

### 2.20 Adverse event reporting and safety

All adverse events will be recorded and discussed at weekly safety meetings by at least 2 investigators. If clinically indicated, the nature of the analgesia administered in the study may be revealed. After assessment by the Principle Investigator, any SAEs will be reported to the Ethics Committee in PUMCH. All SAEs will be also reported to the China Food and Drug Administration by the Ethics Committee.

### 2.21 Data analysis

Data distribution will be tested for normality using the Shapiro–Wilk test. Skewed data will be corrected by logit-transformation as appropriate and subsequently examined using parametric test in order to preserve statistical power. For data set that cannot be normalized by logit-transformation, nonparametric test will be applied. Group comparison will be done by Student *t* test and Mann–Whitney *U* test for parametric and nonparametric data, respectively. Pearson Chi-square will be used for categorical variables. In strata where the expected counts are less than 5, Fisher exact test will be used. Univariate logistic regression and simple linear regression will be performed to determine potential predictors associated with binary outcomes and continuous outcomes, respectively. For predictors significantly associated with the outcomes of interest (*P* < .1), they will be further examined for co-linearity using Chi-square test, 0-order Pearson product-moment correlation, or Student *t* test as appropriate. For continuous variables with variable inflation factor greater or equal to 10, only 1 variable will be kept for subsequent analysis. After removing redundant co-variates, multivariate regression models will be built. The goodness-of-fit of the final models will be determined by the Hosmer–Lemeshow test (logistic regression) or *R*^2^ (multiple linear regression), respectively. For time-to-event outcomes, multivariate Cox proportional hazard regression models will be built. The cumulative probability of an outcome event over time will be presented in form of Kaplan–Meier curves.

To determine changes of analgesic consumption and pain score over time between groups, a generalized estimating equation population average regression will be used. To correct for ceiling effect of analgesic consumption on pain score reporting, an empirical logit transformation will be applied in before comparison between groups. For missing data less than 10%, imputation will be performed using median for continuous and ordinal variables. For nominal variables, the most common category will be imputed. If there are more than 10% missing data, multiple imputation will be used. Intention-to-treat and per-protocol analyses will be performed to account for possible missing data. *P*-value less than .05 will be considered as statistical significance. All statistical analyses will be 2-tailed and performed using Statistical Package for Social Sciences (SPSS version 24.0, Chicago, IL).

### 2.22 Ethics and dissemination

The study has been approved by the Ethics Committee of Peking Union Medical College Hospital. This trial is registered with the Chinese Clinical Trial Registry (ChiCTR-IPR-17013094). On completion of the trial, the findings will be analyzed and tabulated. Results of the trial will be submitted for publication in a peer-reviewed journal.

## 3 Discussion

To maximize postoperative pain relief, enhance patient satisfaction, and facilitate rehabilitation after surgery, pain management is indispensably important. Multimodal analgesia for pain management after surgery has been shown effective.^[5]^ However, no optimal regimen for pain control after spine surgery has been found. Therefore, we design a novel multimodal analgesic regimen for wound infiltration. Ropivacaine is a propyl analog of bupivacaine with longer duration of action and reversibly inhibits sodium ion influx in nerve fibers. It has lesser proclivity to penetrate large myelinated motor fibers compared with bupivacaine due to its low lipophilic nature. This is responsible for differential sensory blockade of ropivacaine with less central nervous system and cardiac toxicity compared with bupivacaine.^[18]^ Parecoxib is a selective COX-2 inhibitor shown to be as effective as traditional NSAIDs as an analgesic for acute postoperative pain whilst having fewer gastrointestinal side effects than traditional NSAIDs, such as diclofenac and ibuprofen.^[19–21]^ Moreover, parecoxib has no effects on serum thromboxane and platelet functions, suggesting that it may be an effective postoperative analgesic.^[22]^ Betamethasone produce strong anti-inflammatory action. This study hypothesized that cocktail analgesic injection could provide superior postoperative analgesia after lumbar spinal fusion surgery. In addition, this route of administration is safe, convenient, and general-purpose way in practice. To date; however, no comparative data are available. Therefore, we believe that this study will provide some insight into perioperative multimodal analgesia, which helps to improve perioperative clinical practice.

## Author contributions

**Data curation:** Zhinan Ren, Lin Sheng, Xin Chen, William Ka Kei Wu, Matthew T.V. Chan, Jeffery Ho.

**Formal analysis:** Derong Xu.

**Software:** Zheng Li.

**Supervision:** Shugang li.

**Writing – original draft:** Zhinan Ren.

**Writing – review and editing:** Shugang li.

## References

[1] Bianconi M Ferraro L Ricci R The pharmacokinetics and efficacy of ropivacaine continuous wound instillation after spine fusion surgery. Anesth Analg 2004;98:166–72. 14693613 10.1213/01.ANE.0000093310.47375.44

[2] Rusy LM Hainsworth KR Nelson TJ Gabapentin use in pediatric spinal fusion patients: a randomized, double-blind, controlled trial. Anesth Analg 2010;110:1393–8. 20418301 10.1213/ANE.0b013e3181d41dc2

[3] Mathiesen O Dahl B Thomsen BA A comprehensive multimodal pain treatment reduces opioid consumption after multilevel spine surgery. Eur Spine J 2013;22:2089–96. 23681498 10.1007/s00586-013-2826-1 PMC3777071

[4] Yukawa Y Kato F Ito K A prospective randomized study of preemptive analgesia for postoperative pain in the patients undergoing posterior lumbar interbody fusion: continuous subcutaneous morphine, continuous epidural morphine, and diclofenac sodium. Spine (Phila Pa 1976) 2005;30:2357–61. 16261108 10.1097/01.brs.0000184377.31427.fa

[5] American Society of Anesthesiologists Task Force on Acute Pain Management. Practice guidelines for acute pain management in the perioperative setting: an updated report by the American Society of Anesthesiologists Task Force on Acute Pain Management. Anesthesiology 2012;116:248–73. 22227789 10.1097/ALN.0b013e31823c1030

[6] Davies AF Segar EP Murdoch J Epidural infusion or combined femoral and sciatic nerve blocks as perioperative analgesia for knee arthroplasty. Br J Anaesth 2004;93:368–74. 15247111 10.1093/bja/aeh224

[7] Ilfeld BM Gearen PF Enneking FK Total knee arthroplasty as an overnight-stay procedure using continuous femoral nerve blocks at home: a prospective feasibility study. Anesth Analg 2006;102:87–90. 16368810 10.1213/01.ane.0000189562.86969.9f

[8] Singelyn FJ Deyaert M Joris D Effects of intravenous patient-controlled analgesia with morphine, continuous epidural analgesia, and continuous three-in-one block on postoperative pain and knee rehabilitation after unilateral total knee arthroplasty. Anesth Analg 1998;87:88–92. 9661552 10.1097/00000539-199807000-00019

[9] Busch CA Shore BJ Bhandari R Efficacy of periarticular multimodal drug injection in total knee arthroplasty. A randomized trial. J Bone Joint Surg Am 2006;88:959–63. 16651569 10.2106/JBJS.E.00344

[10] Vendittoli PA Makinen P Drolet P A multimodal analgesia protocol for total knee arthroplasty. A randomized, controlled study. J Bone Joint Surg Am 2006;88:282–9. 16452738 10.2106/JBJS.E.00173

[11] Goyal N McKenzie J Sharkey PF The 2012 Chitranjan Ranawat award: intraarticular analgesia after TKA reduces pain: a randomized, double-blinded, placebo-controlled, prospective study. Clin Orthop Relat Res 2013;471:64–75. 23011843 10.1007/s11999-012-2596-9 PMC3528916

[12] Bianconi M Ferraro L Traina GC Pharmacokinetics and efficacy of ropivacaine continuous wound instillation after joint replacement surgery. Br J Anaesth 2003;91:830–5. 14633754 10.1093/bja/aeg277

[13] Isaac D Falode T Liu P Accelerated rehabilitation after total knee replacement. Knee 2005;12:346–50. 16019214 10.1016/j.knee.2004.11.007

[14] Rasmussen S Kramhoft MU Sperling KP Increased flexion and reduced hospital stay with continuous intraarticular morphine and ropivacaine after primary total knee replacement: open intervention study of efficacy and safety in 154 patients. Acta Orthop Scand 2004;75:606–9. 15513495 10.1080/00016470410001501

[15] Kelly AM The minimum clinically significant difference in visual analogue scale pain score does not differ with severity of pain. Emerg Med J 2001;18:205–7. 11354213 10.1136/emj.18.3.205 PMC1725574

[16] Todd KH Clinical versus statistical significance in the assessment of pain relief. Ann Emerg Med 1996;27:439–41. 8604855 10.1016/s0196-0644(96)70226-3

[17] Kelly AM Does the clinically significant difference in visual analog scale pain scores vary with gender, age, or cause of pain? Acad Emerg Med 1998;5:1086–90. 9835471 10.1111/j.1553-2712.1998.tb02667.x

[18] Knudsen K Beckman SM Blomberg S Central nervous and cardiovascular effects of i.v. infusions of ropivacaine, bupivacaine and placebo in volunteers. Br J Anaesth 1997;78:507–14. 9175963 10.1093/bja/78.5.507

[19] Mu DL Zhang DZ Wang DX Parecoxib supplementation to morphine analgesia decreases incidence of delirium in elderly patients after hip or knee replacement surgery: a randomized controlled trial. Anesth Analg 2017;124:1992–2000. 28525512 10.1213/ANE.0000000000002095

[20] Diaz-Borjon E Torres-Gomez A Essex MN Parecoxib provides analgesic and opioid-sparing effects following major orthopedic surgery: a subset analysis of a randomized, placebo-controlled clinical trial. Pain Ther 2017;6:61–72. 28255955 10.1007/s40122-017-0066-5 PMC5447543

[21] Parsons B Zhu Q Xie L Effects of parecoxib on postoperative pain and opioid-related symptoms following gynecologic surgery. J Pain Res 2016;9:1101–7. 27932894 10.2147/JPR.S111733 PMC5135478

[22] Essex MN Cheung R Li C Safety of parecoxib when used for more than 3 days for the management of postoperative pain. Pain Manag 2017;7:383–9. 28589749 10.2217/pmt-2017-0017

